# Supplementary material for: Modeling glioblastoma heterogeneity as a dynamic network of cell states
Source: Mol Syst Biol. 2021 Sep 16;17(9):e10105. doi: 10.15252/msb.202010105 (PMC8444284; doi:10.15252/msb.202010105)
Supplement: Supplementary file 5 — Source Data for Figure 3 [file MSB-17-e10105-s001.zip › Figure3A_sourcedata/GSEA_3065/nosigdb_state2.GseaPreranked.1622718419215/FELDMAN_2020_G2-M.html]

Details for gene set FELDMAN\_2020\_G2-M[GSEA]

|  || Dataset | state2 |
| Phenotype | NoPhenotypeAvailable |
| Upregulated in class | na\_pos |
| GeneSet | FELDMAN\_2020\_G2-M |
| Enrichment Score (ES) | 0.7279957 |
| Normalized Enrichment Score (NES) | 2.71466 |
| Nominal p-value | 0.0 |
| FDR q-value | 0.0 |
| FWER p-Value | 0.0 |
Table: GSEA Results Summary

  

Fig 1: Enrichment plot: FELDMAN\_2020\_G2-M      
 Profile of the Running ES Score & Positions of GeneSet Members on the Rank Ordered List

  

| PROBE | GENE SYMBOL | GENE\_TITLE | RANK IN GENE LIST | RANK METRIC SCORE | RUNNING ES | CORE ENRICHMENT || 1 | HMGB2 |  |  | 4 | 0.823 | 0.0107 | Yes |
| 2 | H2AFZ |  |  | 8 | 0.735 | 0.0203 | Yes |
| 3 | UBE2C |  |  | 9 | 0.704 | 0.0299 | Yes |
| 4 | H2AFX |  |  | 10 | 0.675 | 0.0390 | Yes |
| 5 | UBE2T |  |  | 12 | 0.643 | 0.0476 | Yes |
| 6 | TOP2A |  |  | 15 | 0.596 | 0.0554 | Yes |
| 7 | MKI67 |  |  | 17 | 0.590 | 0.0633 | Yes |
| 8 | CDK1 |  |  | 18 | 0.577 | 0.0711 | Yes |
| 9 | UBE2S |  |  | 19 | 0.574 | 0.0789 | Yes |
| 10 | MAD2L1 |  |  | 20 | 0.563 | 0.0865 | Yes |
| 11 | RRM2 |  |  | 22 | 0.556 | 0.0940 | Yes |
| 12 | CKS1B |  |  | 23 | 0.554 | 0.1015 | Yes |
| 13 | HMGB1 |  |  | 25 | 0.549 | 0.1088 | Yes |
| 14 | NUDT1 |  |  | 26 | 0.541 | 0.1161 | Yes |
| 15 | CKS2 |  |  | 28 | 0.532 | 0.1232 | Yes |
| 16 | TPX2 |  |  | 29 | 0.531 | 0.1304 | Yes |
| 17 | ESCO2 |  |  | 30 | 0.530 | 0.1376 | Yes |
| 18 | DEK |  |  | 31 | 0.526 | 0.1447 | Yes |
| 19 | STMN1 |  |  | 33 | 0.520 | 0.1516 | Yes |
| 20 | CENPF |  |  | 34 | 0.518 | 0.1586 | Yes |
| 21 | HMGN2 |  |  | 35 | 0.512 | 0.1656 | Yes |
| 22 | RPA3 |  |  | 36 | 0.511 | 0.1725 | Yes |
| 23 | SMC2 |  |  | 39 | 0.493 | 0.1790 | Yes |
| 24 | DBF4 |  |  | 41 | 0.492 | 0.1855 | Yes |
| 25 | SMC4 |  |  | 42 | 0.489 | 0.1921 | Yes |
| 26 | TMPO |  |  | 43 | 0.489 | 0.1988 | Yes |
| 27 | CENPK |  |  | 44 | 0.488 | 0.2054 | Yes |
| 28 | ZWINT |  |  | 45 | 0.488 | 0.2120 | Yes |
| 29 | PBK |  |  | 46 | 0.484 | 0.2185 | Yes |
| 30 | NUSAP1 |  |  | 47 | 0.483 | 0.2250 | Yes |
| 31 | ANP32B |  |  | 48 | 0.474 | 0.2315 | Yes |
| 32 | PTMA |  |  | 49 | 0.474 | 0.2379 | Yes |
| 33 | KPNA2 |  |  | 50 | 0.472 | 0.2443 | Yes |
| 34 | TUBA1B |  |  | 52 | 0.462 | 0.2504 | Yes |
| 35 | CENPM |  |  | 54 | 0.460 | 0.2565 | Yes |
| 36 | TK1 |  |  | 56 | 0.456 | 0.2626 | Yes |
| 37 | NCAPG |  |  | 60 | 0.445 | 0.2683 | Yes |
| 38 | BIRC5 |  |  | 61 | 0.443 | 0.2743 | Yes |
| 39 | PTTG1 |  |  | 63 | 0.439 | 0.2801 | Yes |
| 40 | GMNN |  |  | 64 | 0.435 | 0.2860 | Yes |
| 41 | RANBP1 |  |  | 65 | 0.430 | 0.2918 | Yes |
| 42 | FBXO5 |  |  | 67 | 0.420 | 0.2974 | Yes |
| 43 | CCNB1 |  |  | 68 | 0.420 | 0.3031 | Yes |
| 44 | LMNB1 |  |  | 69 | 0.417 | 0.3087 | Yes |
| 45 | CENPH |  |  | 71 | 0.412 | 0.3142 | Yes |
| 46 | LSM4 |  |  | 73 | 0.410 | 0.3197 | Yes |
| 47 | SNRPD1 |  |  | 74 | 0.408 | 0.3252 | Yes |
| 48 | LSM3 |  |  | 75 | 0.407 | 0.3307 | Yes |
| 49 | NUCKS1 |  |  | 78 | 0.404 | 0.3359 | Yes |
| 50 | TUBB |  |  | 81 | 0.397 | 0.3411 | Yes |
| 51 | H2AFV |  |  | 82 | 0.397 | 0.3465 | Yes |
| 52 | KIF4A |  |  | 84 | 0.393 | 0.3517 | Yes |
| 53 | LSM5 |  |  | 85 | 0.393 | 0.3570 | Yes |
| 54 | CENPN |  |  | 87 | 0.390 | 0.3622 | Yes |
| 55 | GTSE1 |  |  | 89 | 0.389 | 0.3673 | Yes |
| 56 | PHF19 |  |  | 93 | 0.380 | 0.3721 | Yes |
| 57 | DTYMK |  |  | 94 | 0.379 | 0.3773 | Yes |
| 58 | CDKN3 |  |  | 97 | 0.376 | 0.3821 | Yes |
| 59 | RAN |  |  | 98 | 0.375 | 0.3872 | Yes |
| 60 | SPC25 |  |  | 104 | 0.372 | 0.3917 | Yes |
| 61 | CENPE |  |  | 105 | 0.372 | 0.3967 | Yes |
| 62 | CACYBP |  |  | 109 | 0.366 | 0.4014 | Yes |
| 63 | RHEB |  |  | 110 | 0.362 | 0.4063 | Yes |
| 64 | ANLN |  |  | 111 | 0.358 | 0.4111 | Yes |
| 65 | NDC80 |  |  | 115 | 0.354 | 0.4156 | Yes |
| 66 | KIF23 |  |  | 117 | 0.351 | 0.4202 | Yes |
| 67 | ASPM |  |  | 122 | 0.342 | 0.4244 | Yes |
| 68 | CDKN2C |  |  | 123 | 0.341 | 0.4290 | Yes |
| 69 | TUBB4B |  |  | 125 | 0.340 | 0.4335 | Yes |
| 70 | CCDC34 |  |  | 127 | 0.339 | 0.4380 | Yes |
| 71 | PTGES3 |  |  | 128 | 0.336 | 0.4426 | Yes |
| 72 | KIF11 |  |  | 129 | 0.335 | 0.4471 | Yes |
| 73 | TACC3 |  |  | 130 | 0.334 | 0.4516 | Yes |
| 74 | SNRPB |  |  | 132 | 0.332 | 0.4560 | Yes |
| 75 | MELK |  |  | 133 | 0.332 | 0.4605 | Yes |
| 76 | POC1A |  |  | 134 | 0.332 | 0.4650 | Yes |
| 77 | CCNA2 |  |  | 135 | 0.331 | 0.4695 | Yes |
| 78 | ANP32E |  |  | 136 | 0.330 | 0.4740 | Yes |
| 79 | SRSF3 |  |  | 140 | 0.327 | 0.4781 | Yes |
| 80 | MIS18A |  |  | 142 | 0.322 | 0.4823 | Yes |
| 81 | CALM2 |  |  | 143 | 0.322 | 0.4867 | Yes |
| 82 | ARHGAP11A |  |  | 145 | 0.321 | 0.4909 | Yes |
| 83 | RAD21 |  |  | 147 | 0.316 | 0.4951 | Yes |
| 84 | CDCA2 |  |  | 150 | 0.309 | 0.4990 | Yes |
| 85 | CKAP2 |  |  | 151 | 0.308 | 0.5032 | Yes |
| 86 | CBX3 |  |  | 152 | 0.308 | 0.5074 | Yes |
| 87 | KIF22 |  |  | 154 | 0.306 | 0.5114 | Yes |
| 88 | CEP55 |  |  | 158 | 0.306 | 0.5152 | Yes |
| 89 | CDCA3 |  |  | 160 | 0.304 | 0.5192 | Yes |
| 90 | TMEM106C |  |  | 162 | 0.303 | 0.5232 | Yes |
| 91 | AURKB |  |  | 163 | 0.303 | 0.5273 | Yes |
| 92 | CHCHD2 |  |  | 165 | 0.302 | 0.5313 | Yes |
| 93 | SLC25A5 |  |  | 168 | 0.299 | 0.5351 | Yes |
| 94 | CMC2 |  |  | 170 | 0.297 | 0.5391 | Yes |
| 95 | ECT2 |  |  | 171 | 0.297 | 0.5431 | Yes |
| 96 | MZT1 |  |  | 174 | 0.295 | 0.5469 | Yes |
| 97 | HMGN1 |  |  | 182 | 0.293 | 0.5501 | Yes |
| 98 | DDX39A |  |  | 183 | 0.293 | 0.5540 | Yes |
| 99 | NUF2 |  |  | 188 | 0.287 | 0.5575 | Yes |
| 100 | CCNB2 |  |  | 190 | 0.286 | 0.5612 | Yes |
| 101 | CDC20 |  |  | 193 | 0.284 | 0.5649 | Yes |
| 102 | DEPDC1 |  |  | 196 | 0.283 | 0.5685 | Yes |
| 103 | HMGB3 |  |  | 200 | 0.282 | 0.5720 | Yes |
| 104 | FOXM1 |  |  | 202 | 0.282 | 0.5757 | Yes |
| 105 | CYCS |  |  | 207 | 0.279 | 0.5790 | Yes |
| 106 | KIF20B |  |  | 208 | 0.278 | 0.5828 | Yes |
| 107 | HJURP |  |  | 209 | 0.277 | 0.5865 | Yes |
| 108 | HNRNPDL |  |  | 220 | 0.271 | 0.5891 | Yes |
| 109 | SHCBP1 |  |  | 231 | 0.262 | 0.5916 | Yes |
| 110 | CENPA |  |  | 238 | 0.259 | 0.5944 | Yes |
| 111 | ILF2 |  |  | 240 | 0.258 | 0.5978 | Yes |
| 112 | CCDC167 |  |  | 243 | 0.255 | 0.6010 | Yes |
| 113 | PPP1CC |  |  | 246 | 0.253 | 0.6042 | Yes |
| 114 | KIF2C |  |  | 252 | 0.251 | 0.6071 | Yes |
| 115 | SPC24 |  |  | 256 | 0.248 | 0.6101 | Yes |
| 116 | MIS18BP1 |  |  | 257 | 0.246 | 0.6135 | Yes |
| 117 | TUBG1 |  |  | 262 | 0.246 | 0.6163 | Yes |
| 118 | PSMC3 |  |  | 275 | 0.242 | 0.6183 | Yes |
| 119 | SAE1 |  |  | 278 | 0.242 | 0.6214 | Yes |
| 120 | CCT5 |  |  | 279 | 0.242 | 0.6246 | Yes |
| 121 | PRR11 |  |  | 285 | 0.238 | 0.6273 | Yes |
| 122 | TRIP13 |  |  | 286 | 0.237 | 0.6305 | Yes |
| 123 | KIF14 |  |  | 302 | 0.231 | 0.6320 | Yes |
| 124 | BUB3 |  |  | 311 | 0.228 | 0.6342 | Yes |
| 125 | C12orf75 |  |  | 316 | 0.227 | 0.6369 | Yes |
| 126 | HNRNPA3 |  |  | 320 | 0.226 | 0.6396 | Yes |
| 127 | DIAPH3 |  |  | 321 | 0.226 | 0.6426 | Yes |
| 128 | CKAP2L |  |  | 327 | 0.224 | 0.6451 | Yes |
| 129 | MXD3 |  |  | 328 | 0.224 | 0.6482 | Yes |
| 130 | RACGAP1 |  |  | 333 | 0.222 | 0.6507 | Yes |
| 131 | BUB1 |  |  | 335 | 0.222 | 0.6536 | Yes |
| 132 | MRPL51 |  |  | 349 | 0.217 | 0.6551 | Yes |
| 133 | HMMR |  |  | 358 | 0.215 | 0.6572 | Yes |
| 134 | PLK1 |  |  | 364 | 0.213 | 0.6595 | Yes |
| 135 | DLGAP5 |  |  | 371 | 0.211 | 0.6617 | Yes |
| 136 | CENPW |  |  | 373 | 0.211 | 0.6645 | Yes |
| 137 | AURKA |  |  | 375 | 0.210 | 0.6672 | Yes |
| 138 | GLRX5 |  |  | 386 | 0.207 | 0.6689 | Yes |
| 139 | RBM8A |  |  | 403 | 0.199 | 0.6699 | Yes |
| 140 | KIF18A |  |  | 419 | 0.195 | 0.6709 | Yes |
| 141 | NCAPD2 |  |  | 434 | 0.193 | 0.6719 | Yes |
| 142 | TROAP |  |  | 435 | 0.193 | 0.6746 | Yes |
| 143 | SPAG5 |  |  | 439 | 0.192 | 0.6768 | Yes |
| 144 | CALM3 |  |  | 446 | 0.191 | 0.6787 | Yes |
| 145 | EXOSC8 |  |  | 456 | 0.189 | 0.6803 | Yes |
| 146 | SPDL1 |  |  | 459 | 0.189 | 0.6827 | Yes |
| 147 | MAD2L2 |  |  | 460 | 0.189 | 0.6852 | Yes |
| 148 | RTKN2 |  |  | 463 | 0.188 | 0.6876 | Yes |
| 149 | DCP2 |  |  | 474 | 0.184 | 0.6890 | Yes |
| 150 | KNSTRN |  |  | 483 | 0.183 | 0.6906 | Yes |
| 151 | TCP1 |  |  | 490 | 0.182 | 0.6924 | Yes |
| 152 | HNRNPH3 |  |  | 504 | 0.180 | 0.6934 | Yes |
| 153 | DEPDC1B |  |  | 505 | 0.179 | 0.6958 | Yes |
| 154 | LDHA |  |  | 506 | 0.179 | 0.6982 | Yes |
| 155 | CBX1 |  |  | 509 | 0.179 | 0.7004 | Yes |
| 156 | HNRNPA2B1 |  |  | 510 | 0.178 | 0.7029 | Yes |
| 157 | PRC1 |  |  | 514 | 0.178 | 0.7049 | Yes |
| 158 | LRR1 |  |  | 516 | 0.178 | 0.7072 | Yes |
| 159 | BCL2L12 |  |  | 517 | 0.178 | 0.7097 | Yes |
| 160 | CDC25B |  |  | 519 | 0.178 | 0.7120 | Yes |
| 161 | SNRNP25 |  |  | 525 | 0.177 | 0.7138 | Yes |
| 162 | VDAC3 |  |  | 528 | 0.176 | 0.7160 | Yes |
| 163 | BUB1B |  |  | 572 | 0.168 | 0.7135 | Yes |
| 164 | KIFC1 |  |  | 577 | 0.167 | 0.7154 | Yes |
| 165 | PARPBP |  |  | 594 | 0.164 | 0.7158 | Yes |
| 166 | CKAP5 |  |  | 596 | 0.164 | 0.7180 | Yes |
| 167 | HSP90AA1 |  |  | 600 | 0.164 | 0.7198 | Yes |
| 168 | KIF5B |  |  | 604 | 0.163 | 0.7217 | Yes |
| 169 | TUBB6 |  |  | 622 | 0.160 | 0.7220 | Yes |
| 170 | CDCA8 |  |  | 634 | 0.158 | 0.7230 | Yes |
| 171 | TIMM10 |  |  | 639 | 0.158 | 0.7247 | Yes |
| 172 | S100B |  |  | 688 | 0.151 | 0.7215 | Yes |
| 173 | EIF5 |  |  | 699 | 0.150 | 0.7224 | Yes |
| 174 | MT2A |  |  | 731 | 0.146 | 0.7210 | Yes |
| 175 | NUDCD2 |  |  | 751 | 0.143 | 0.7209 | Yes |
| 176 | FAM83D |  |  | 759 | 0.143 | 0.7220 | Yes |
| 177 | SSNA1 |  |  | 763 | 0.142 | 0.7236 | Yes |
| 178 | SKA3 |  |  | 790 | 0.139 | 0.7227 | Yes |
| 179 | STIP1 |  |  | 794 | 0.139 | 0.7242 | Yes |
| 180 | NEK2 |  |  | 805 | 0.138 | 0.7250 | Yes |
| 181 | FABP7 |  |  | 810 | 0.137 | 0.7264 | Yes |
| 182 | H3F3B |  |  | 829 | 0.135 | 0.7263 | Yes |
| 183 | TTK |  |  | 831 | 0.135 | 0.7280 | Yes |
| 184 | RHNO1 |  |  | 857 | 0.132 | 0.7270 | No |
| 185 | HNRNPA1 |  |  | 876 | 0.130 | 0.7268 | No |
| 186 | SFPQ |  |  | 967 | 0.122 | 0.7187 | No |
| 187 | RPL31 |  |  | 1000 | 0.120 | 0.7168 | No |
| 188 | PSRC1 |  |  | 1023 | 0.118 | 0.7160 | No |
| 189 | KIF20A |  |  | 1094 | 0.112 | 0.7099 | No |
| 190 | RBMX |  |  | 1186 | 0.105 | 0.7013 | No |
| 191 | HP1BP3 |  |  | 1249 | 0.102 | 0.6959 | No |
| 192 | CLIC1 |  |  | 1291 | 0.099 | 0.6928 | No |
| 193 | NUP35 |  |  | 1292 | 0.099 | 0.6941 | No |
| 194 | HES6 |  |  | 1305 | 0.098 | 0.6941 | No |
| 195 | CDC27 |  |  | 1319 | 0.097 | 0.6940 | No |
| 196 | GAS2L3 |  |  | 1326 | 0.096 | 0.6947 | No |
| 197 | MPHOSPH9 |  |  | 1356 | 0.094 | 0.6928 | No |
| 198 | PRPSAP1 |  |  | 1394 | 0.092 | 0.6900 | No |
| 199 | DCXR |  |  | 1477 | 0.088 | 0.6822 | No |
| 200 | CCDC18 |  |  | 1491 | 0.087 | 0.6820 | No |
| 201 | MORF4L2 |  |  | 1548 | 0.084 | 0.6770 | No |
| 202 | RNASEH2A |  |  | 1579 | 0.082 | 0.6748 | No |
| 203 | REEP4 |  |  | 1734 | 0.076 | 0.6590 | No |
| 204 | PRDX1 |  |  | 1763 | 0.074 | 0.6570 | No |
| 205 | RNF26 |  |  | 1787 | 0.073 | 0.6555 | No |
| 206 | RUVBL2 |  |  | 2141 | 0.060 | 0.6177 | No |
| 207 | GGH |  |  | 2149 | 0.059 | 0.6177 | No |
| 208 | OIP5 |  |  | 2175 | 0.058 | 0.6158 | No |
| 209 | METRN |  |  | 2177 | 0.058 | 0.6165 | No |
| 210 | EMC9 |  |  | 2216 | 0.057 | 0.6131 | No |
| 211 | RPL34 |  |  | 2224 | 0.056 | 0.6131 | No |
| 212 | FTH1 |  |  | 2254 | 0.055 | 0.6107 | No |
| 213 | C4orf48 |  |  | 2255 | 0.055 | 0.6114 | No |
| 214 | OST4 |  |  | 2267 | 0.055 | 0.6109 | No |
| 215 | C4orf3 |  |  | 2337 | 0.052 | 0.6041 | No |
| 216 | ISCA2 |  |  | 2368 | 0.052 | 0.6015 | No |
| 217 | G2E3 |  |  | 2405 | 0.051 | 0.5983 | No |
| 218 | ARL6IP1 |  |  | 2421 | 0.050 | 0.5973 | No |
| 219 | COMMD6 |  |  | 2534 | 0.047 | 0.5857 | No |
| 220 | FOSL1 |  |  | 2584 | 0.045 | 0.5810 | No |
| 221 | GLIPR2 |  |  | 2669 | 0.043 | 0.5724 | No |
| 222 | TMEM237 |  |  | 2862 | 0.037 | 0.5519 | No |
| 223 | ADA |  |  | 3009 | 0.034 | 0.5364 | No |
| 224 | PDLIM7 |  |  | 3015 | 0.033 | 0.5363 | No |
| 225 | TNFAIP8L1 |  |  | 3154 | 0.031 | 0.5216 | No |
| 226 | LIMA1 |  |  | 3279 | 0.028 | 0.5085 | No |
| 227 | GOT1 |  |  | 3561 | 0.023 | 0.4781 | No |
| 228 | RAB13 |  |  | 3928 | 0.017 | 0.4383 | No |
| 229 | SHISA4 |  |  | 4125 | 0.014 | 0.4171 | No |
| 230 | HMGA1 |  |  | 4135 | 0.013 | 0.4163 | No |
| 231 | FAM162A |  |  | 4622 | 0.005 | 0.3633 | No |
| 232 | RBP1 |  |  | 4975 | 0.000 | 0.3248 | No |
| 233 | GPSM2 |  |  | 5127 | -0.002 | 0.3083 | No |
| 234 | HSD17B11 |  |  | 5658 | -0.011 | 0.2506 | No |
| 235 | TXNDC12 |  |  | 5696 | -0.011 | 0.2467 | No |
| 236 | PIF1 |  |  | 5869 | -0.014 | 0.2281 | No |
| 237 | HOXB4 |  |  | 6621 | -0.028 | 0.1464 | No |
| 238 | EEF2 |  |  | 6630 | -0.028 | 0.1459 | No |
| 239 | EEF1A1 |  |  | 6690 | -0.029 | 0.1398 | No |
| 240 | TCEAL8 |  |  | 6716 | -0.030 | 0.1375 | No |
| 241 | PDLIM4 |  |  | 6744 | -0.030 | 0.1350 | No |
| 242 | BEX4 |  |  | 6903 | -0.034 | 0.1182 | No |
| 243 | LBR |  |  | 7064 | -0.038 | 0.1012 | No |
| 244 | FTL |  |  | 7693 | -0.056 | 0.0333 | No |
| 245 | BTG1 |  |  | 8156 | -0.075 | -0.0161 | No |
| 246 | MEST |  |  | 8534 | -0.099 | -0.0560 | No |
| 247 | PLD3 |  |  | 8576 | -0.102 | -0.0591 | No |
| 248 | FILIP1L |  |  | 8586 | -0.102 | -0.0587 | No |
| 249 | DDIT4 |  |  | 8601 | -0.103 | -0.0588 | No |
| 250 | CNIH4 |  |  | 8611 | -0.104 | -0.0584 | No |
| 251 | LMNA |  |  | 8636 | -0.106 | -0.0596 | No |
| 252 | MAP3K7CL |  |  | 8672 | -0.109 | -0.0619 | No |
| 253 | CST3 |  |  | 8712 | -0.112 | -0.0647 | No |
| 254 | IFITM3 |  |  | 8720 | -0.113 | -0.0639 | No |
| 255 | TP53I3 |  |  | 8860 | -0.131 | -0.0773 | No |
| 256 | PLP1 |  |  | 8931 | -0.139 | -0.0831 | No |
| 257 | FGFBP3 |  |  | 8960 | -0.143 | -0.0842 | No |
| 258 | UBC |  |  | 8968 | -0.144 | -0.0830 | No |
| 259 | PTN |  |  | 8989 | -0.147 | -0.0832 | No |
| 260 | TTYH1 |  |  | 9029 | -0.154 | -0.0854 | No |
| 261 | PLIN3 |  |  | 9043 | -0.157 | -0.0847 | No |
| 262 | SOX4 |  |  | 9063 | -0.160 | -0.0846 | No |
| 263 | EIF4A2 |  |  | 9067 | -0.160 | -0.0828 | No |
| 264 | BEX1 |  |  | 9079 | -0.162 | -0.0818 | No |
| 265 | CDC42EP3 |  |  | 9126 | -0.171 | -0.0845 | No |
| 266 | PRCP |  |  | 9153 | -0.178 | -0.0849 | No |
| 267 | ATP6V0E1 |  |  | 9173 | -0.182 | -0.0845 | No |
| 268 | SCRG1 |  |  | 9200 | -0.191 | -0.0848 | No |
| 269 | SAT1 |  |  | 9279 | -0.224 | -0.0903 | No |
| 270 | IGFBP7 |  |  | 9295 | -0.229 | -0.0888 | No |
| 271 | CDKN1A |  |  | 9301 | -0.230 | -0.0862 | No |
| 272 | PCSK1N |  |  | 9321 | -0.247 | -0.0850 | No |
| 273 | TM7SF2 |  |  | 9330 | -0.252 | -0.0824 | No |
| 274 | NOVA1 |  |  | 9348 | -0.272 | -0.0806 | No |
| 275 | PTPRZ1 |  |  | 9351 | -0.272 | -0.0772 | No |
| 276 | PSAP |  |  | 9363 | -0.294 | -0.0744 | No |
| 277 | SPARC |  |  | 9384 | -0.332 | -0.0721 | No |
| 278 | MIAT |  |  | 9386 | -0.332 | -0.0677 | No |
| 279 | GPM6B |  |  | 9389 | -0.344 | -0.0632 | No |
| 280 | CALD1 |  |  | 9391 | -0.348 | -0.0586 | No |
| 281 | ITM2B |  |  | 9399 | -0.371 | -0.0544 | No |
| 282 | CTGF |  |  | 9400 | -0.375 | -0.0493 | No |
| 283 | TNC |  |  | 9403 | -0.383 | -0.0443 | No |
| 284 | GLIPR1 |  |  | 9404 | -0.387 | -0.0391 | No |
| 285 | IGFBP3 |  |  | 9424 | -0.502 | -0.0344 | No |
| 286 | CLU |  |  | 9430 | -0.547 | -0.0275 | No |
| 287 | DCBLD2 |  |  | 9431 | -0.558 | -0.0200 | No |
| 288 | MALAT1 |  |  | 9436 | -0.735 | -0.0105 | No |
| 289 | IGFBP5 |  |  | 9438 | -0.797 | 0.0002 | No |
Table: GSEA details [plain text format]

  

Fig 2: FELDMAN\_2020\_G2-M: Random ES distribution      
 Gene set null distribution of ES for **FELDMAN\_2020\_G2-M**
